# “If I Were Nick”: Men’s Responses to an Interactive Video Drama Series to Support Smoking Cessation

**DOI:** 10.2196/jmir.4491

**Published:** 2015-08-10

**Authors:** Joan L Bottorff, Gayl Sarbit, John L Oliffe, Mary T Kelly, Maria Lohan, Sean Stolp, Paul Sharp

**Affiliations:** ^1^ School of Nursing Faculty of Health and Social Development University of British Columbia Kelowna, BC Canada; ^2^ Faculty of Health Sciences Australian Catholic University Melbourne Australia; ^3^ Institute for Healthy Living and Chronic Disease Prevention University of British Columbia Kelowna, BC Canada; ^4^ School of Nursing, Faculty of Applied Science University of British Columbia Vancouver, BC Canada; ^5^ School of Nursing and Midwifery Queen's University Belfast Belfast Ireland

**Keywords:** smoking cessation, tobacco use, technology, interactive video drama, self-efficacy

## Abstract

**Background:**

Men continue to smoke in greater numbers than women; however, few interventions have been developed and tested to support men’s cessation. Men tend to rely on quitting strategies associated with stereotypical manliness, such as willpower, stoicism, and independence, but they may lack the self-efficacy skills required to sustain a quit. In this paper, we describe the development of and reception to an interactive video drama (IVD) series, composed of 7 brief scenarios, to support and strengthen men’s smoking cessation efforts. The value of IVD in health promotion is predicated on the evidence that viewers engage with the material when they are presented characters with whom they can personally identify. The video dramatizes the challenges unfolding in the life of the main character, Nick, on the first day of his quit and models the skills necessary to embark upon a sustainable quit.

**Objective:**

The objective was to describe men’s responses to the *If I were Nick* IVD series as part of a study of QuitNow Men, an innovative smoking cessation website designed for men. Specific objectives were to explore the resonance of the main character of the IVD series with end-users and explore men’s perceptions of the effectiveness of the IVD series for supporting their quit self-management.

**Methods:**

Seven brief IVD scenarios were developed, filmed with a professional actor, and uploaded to a new online smoking cessation website, QuitNow Men. A sample of 117 men who smoked were recruited into the study and provided baseline data prior to access to the QuitNow Men website for a 6-month period. During this time, 47 men chose to view the IVDs. Their responses to questions about the IVDs were collected in online surveys at 3-month and 6-month time points and analyzed using descriptive statistics.

**Results:**

The majority of participants indicated they related to the main character, Nick. Participants who “strongly agreed” they could relate to Nick perceived significantly higher levels of support from the IVDs than the “neutral” and “disagree” groups (*P*<.001, *d*=2.0, *P*<.001, *d*=3.1). The “agree” and “neutral” groups were significantly higher on rated support from the videos than the “disagree” (*P*<.001, *d*=2.2, *P*=.01, *d*=1.5). Participants’ perception of the main character was independent of participant age, education attainment, or previous quit attempts.

**Conclusions:**

The findings suggest that IVD interventions may be an important addition to men’s smoking cessation programs. Given that the use of IVD scenarios in health promotion is in its infancy, the positive outcomes from this study signal the potential for IVD and warrant ongoing evaluation in smoking cessation and, more generally, men’s health promotion.

## Introduction

### Background

Health promotion and tobacco control efforts have resulted in a steady decline in the prevalence of cigarette smoking in Canada and the United States over the past 50 years; however, the highest rates of tobacco use continue to be among adult men, which far exceed population averages [1]. To address the high prevalence of smoking among men, novel gender-specific resources are needed [2,3].

Scholars in men’s health have argued that men prefer to self-manage their health, but at the same time, their health behaviors are strongly influenced by socially produced constructs of masculinity [4,5]. In terms of smoking cessation, men tend to rely on quitting strategies associated with stereotypical manliness, such as willpower, stoicism, and independence [6]. Although men often rely on willpower and a “cold turkey” approach to quit smoking [7], it is well established that this is one of the least effective means of smoking cessation [8]. Despite the availability of a range of smoking cessation programs, there are few programs tailored specifically for men [3]. However, emerging evidence related to men’s experiences and preferences with smoking cessation, along with established approaches to supporting behavior change, provide an important foundation for tailored interventions. For example, men’s preferences for self-managing their quit and making their own decisions about when and how to quit have been reported [9]. There is evidence that these gendered preferences can be supported and enhanced by the use of online information and communication technologies in men’s health promotion [10]. In addition, men’s interest in learning about others’ real-life cessation efforts as a way to develop effective plans for quitting and acquiring strategies that they might also use has been described [2]. Information to support anticipatory planning and skill building can raise self-efficacy and increase confidence and the ability to stop smoking. There is agreement that strengthening self-efficacy by garnering skills and confidence to respond to challenging situations, stressful events, and cravings is an important component in providing support for smoking cessation [11]. Tailoring supports for men’s anticipatory planning and skill building related to quitting could, therefore, provide important additions to cessation programs.

Interactive video drama (IVD) is an emergent media tool with potential for the field of smoking cessation interventions for men. The value of IVD is predicated on the evidence that viewers engage with, and are more influenced by, material when they are presented with a character with whom they personally identify. In a feasibility trial conducted in Northern Ireland, Lohan et al [12] developed and are testing a 4-week IVD program for sex education classes in 7 schools with the aim of reducing unintended teenage pregnancy. Based on observational data of over 700 young men, their video intervention is theorized to strengthen the intentions of male teens to avoid unplanned pregnancy by influencing the psychosocial variables involved in sexual behaviors and choices [12]. To date, IVDs have not been utilized or evaluated in smoking cessation programs.

The capacity for IVDs to incorporate multiple features that are important to men, and at the same time, promote anticipatory thinking and model skills associated with strong self-efficacy makes the approach a good fit to support men’s cessation efforts. The drama-based approach, characteristic of IVDs, offers an important way to engage men and maintain their interest by using men’s authentic voices to present challenging situations wherein the main character models change and effective problem solving– such as the decision to stop smoking and maintain a quit. IVDs also may be effective for men because of the motivational appeal of interactive resources, the popularity of videos with men, the high level of interest in learning about others’ “real life” experiences, and the desire by men to see where they stand in relation to choices made by other men.

As part of a study to design and evaluate an online smoking cessation resource designed specifically for men, QuitNow Men [13], we developed an IVD series, *If I were Nick*, as a resource to support men’s cessation efforts. This IVD series was included on the QuitNow Men website along with a broad array of interactive tools and tips for men interested in quitting smoking, such as a questionnaire to determine their level of nicotine dependence, a calculator to show the financial cost of smoking, expert information and motivational videos, strategies for quitting, and quizzes to test tobacco use knowledge. The IVD provided the opportunity to influence men’s beliefs about quitting through their identification with the central character and dramatized evidenced-based content to strengthen smoking cessation self-efficacy by prompting reflection on smoking triggers, encouraging planning and response strategies for stressors before they arise, and modeling how to incorporate these strategies into daily life to maximize a successful quit. In this paper, we describe the development of this IVD series and report findings related to the resonance of the main character of the IVD series with end-users, and men’s perceptions of the effectiveness of the IVDs for supporting their quit self-management.

### The Framework and Development of If I Were Nick

The *If I Were Nick* videos were developed in accordance with the theoretical framework described in the work of Lohan et al [12] and Aventin et al [14] that blends social theory on gender norms and masculinity with cognitive learning theory and the theory of planned behavior, which have demonstrated that attitudes, perceived control, social context, and intentions influence behavior change [15,16]. A script was written and a story board developed by the team to illustrate high-risk situations that might happen to Nick, a construction worker, during the first day of a quit. Ideas for the high-risk situations were based on focus group data collected with men who smoke [2] and cues for smoking among male smokers that have been identified in the literature [17]. The role of Nick was performed by a professional actor who met the qualifications established by the research team to best engage the target audience: age 25-35 years, genuine and personable on camera, strong male voice, and good range of emotion. Seven scenarios were filmed, each scenario running about one minute. Following each scenario, two online questions prompted viewers to reflect and respond: “How would you feel if you were Nick in this situation?” and “How would you act in this situation in order to remain smoke free?” Viewers were presented with a multiple choice list of 4-6 answer options and asked to click on their selection. The questions were designed to promote anticipatory thinking around smoking cessation and encourage men to consider how they might respond when facing the challenges of quitting. After making their selection, the viewer was able to see how other men responded. Table 1 summarizes the IVD scenarios and reflective questions. Table 2 provides additional details for one scenario from the IVD series, entitled “Morning Routine”, including the response items for the self-reflective and action-oriented questions.

**Table 1:** Story development of the interactive video drama, *If I Were Nick*.

Video scenario	High-risk quit situation	Self-reflective question	Action question
Meet Nick (Multimedia Appendix 1)	At home, Nick tells us he is quitting smoking today and has tried many times in the past. He wants us to understand the experience through his eyes.	If I were Nick, how would I feel on the first day of my quit?	If I were Nick, what tactic would I choose?
Morning Routine (Multimedia Appendix 2)	A flashback in Nick’s mind shows us how the usual morning coffee with a cigarette is the first challenge of the day.	If I were Nick, how would I feel about changing my morning routine?	If I were Nick, what would I change?
On the Road (Multimedia Appendix 3)	Nick pulls into traffic on the way to work and is forced to wait at a red light, which causes him to fidget and feel anxious.	If I were Nick, how would I feel about being in my car without any smokes?	If I were Nick, how would I deal with cravings while driving?
I Need a Break (Multimedia Appendix 4)	On the job site, Nick is conflicted about taking a break with his co-workers, because they all smoke. He worries that if he tells his foreman he’s now a non-smoker, he won’t get as many breaks.	If I were Nick, how would I feel about telling my boss and fellow workers that I’ve quit smoking?	If I were Nick, what could I do at work to avoid temptation?
Out with the Guys (Multimedia Appendix 5)	Nick meets friends at the bar to have a beer and watch the hockey game after work. He’s concerned how his friends will respond to his decision to quit.	If I were Nick, what do I think my friends would say when I tell them I’ve quit smoking?	If I were Nick, how would I respond to pressure from my friends to smoke?
Stressed Out (Multimedia Appendix 6)	Nick enters a convenience store to buy milk and tries to navigate a stressful situation on the telephone while paying the cashier. The interaction rattles him and he notices cigarettes next to the checkout.	If I were Nick, how would I feel about not having a cigarette when I’m stressed?	If I were Nick, how would I stay smoke free in a stressful situation?
On Track (Multimedia Appendix 7)	Nick is lying on the couch thinking about the events of the day and what tomorrow will be like.	If I were Nick, how would I feel after my first quit day?	If I were Nick, how would I keep myself on track?

**Table 2:** Scenario #2 from *If I Were Nick*.

Video segment	High-risk situation	Reflective question	Action question
Morning Routine (Multimedia Appendix 2)	A flashback in Nick’s mind shows us how the usual morning coffee with a cigarette is the first challenge of the day.	If I were Nick, how would I feel about changing my morning routine? (a) I’m proud that I’m making healthy choices for myself, (b) I’ll miss some things I enjoy but I’ll try new things, (c) I feel that I’m strong enough to give it a shot, (d) I’m on the fence about changing my routine, (e) I’m comfortable making changes to my normal routine.	If I were Nick, what would I change? (a) Change the order of my morning routine, (b) Try a different drink, brand of coffee or mug, (c) Move to a new place to drink coffee and eat breakfast, (d) Jump in the shower as soon as I wake up, (e) Do something active in the morning (walk the dog, lift weights at home, do push-ups, hit the gym).

## Methods

### Overview

The current study was conducted using an exploratory descriptive design and received approval from a university ethics review committee.

### Sample

A convenience sample of men who smoked and were interested in quitting were recruited from across Canada using social media and online advertisements. A total of 117 men consented to participate, completed a baseline survey, and were provided access to an online smoking cessation resource, QuitNow Men [13]. As one of the resources on QuitNow Men [13], the streamed IVD series, *If I Were Nick*, was available under a tab labeled “Tactics & Tools”, as one of several resources to support smoking cessation.

The men were invited to use the website independently over a period of 6 months and could choose to use any or all of the resources available. Of the 117 men, 75 completed at least one of the follow-up surveys (response rate=64.1%), and 47 (62.7%) reported that they had viewed one or more of the IVD scenarios. These 47 men provided the data for this study.

### Data Collection

All data collection was completed using an online survey platform at three time points: baseline, 3 months, and 6 months. Participants were provided with two reminders to complete each follow-up survey.

At baseline, demographics were measured including age, marital status, education, and employment as well as participant smoking behaviors. Men’s use of and responses to the IVDs were assessed in the follow-up surveys. A 3-point Likert scale question (“none”, “some”, “all”) was used to assess how many of the Nick video scenarios participants had watched. Participants were asked to respond to the statement “Nick was someone I could relate to” using a 5-point Likert response scale (“strongly disagree” to “strongly agree”). Four 5-point Likert scale questions (“strongly disagree” to “strongly agree”) were included to assess how well the videos supported participants’ self-management by providing them with cognitive and behavioral strategies. The questions were “The videos made me think about issues I hadn’t thought about before”, “As I watched Nick, I thought about how I needed to change my own smoking routines”, “The videos helped me prepare ahead for situations where I might be tempted to smoke”, and “The videos helped me realize that I can be as strong as Nick in staying quit”. These four items were then summed to create an IVD support score*,* rated from 4-20*,* which measured the degree of support the participants derived from viewing the videos. To assess whether the 4 items that were totaled formed a reliable score, Cronbach alpha was computed. The alpha for the four items was .827, indicating that the items formed a scale with good internal consistency. Finally, a 1-item, 5-point Likert scale (“very satisfied” to “very dissatisfied”) assessed participants’ satisfaction with the overall website (ie, “Overall, how satisfied were you with the QuitNow website?”).

### Data Analyses

As men could complete a 3-month survey and a 6-month survey, a hierarchy was developed for data inclusion. If an individual increased the number of IVD scenarios they watched between the 3-month and 6-month time point (ie, “none” to “some,” or “some” to “all”), 6-month data were used. If there was no increase between time points, 3-month data were used, as it was deemed the videos would be more easily recalled at that time. Descriptive statistics were used to analyze the participants’ survey responses and analysis of variance (ANOVA) was used to examine the relationship between levels of relating to Nick and participants’ perceived support from the IVDs. Spearman rho statistic was calculated to assess possible relationships between levels of relating to Nick and participants’ overall website satisfaction. All analyses were conducted using SPSS version 22.

## Results

At baseline, all participants were smokers who were interested in quitting. They smoked on average 14.2 cigarettes a day (SD 7.65) and had been smoking on average for 21.4 years (SD 11.4). Table 3 provides the demographic characteristics of the sample.

**Table 3:** Participant demographics.

		Mean (SD) or n (%)
Age		37.2 (10.3)
**Smoking behavior**		
	Cigarettes smoked per day	14.2 (7.65)
	Years smoking	21.4 (11.4)
**Marital status**		
	Married or common law	25 (53%)
	Single	16 (34%)
	Divorced or separated	6 (13%)
**Education**		
	Attended or completed university	27 (57%)
	Completed high school	9 (19%)
	Completed a non-university degree (technical, trade)	8 (17%)
	Not completed high school	3 (6%)
**Main activity**		
	Work for pay	34 (72%)
	Unemployed	5 (11%)
	Going to school	4 (9%)
	Recovering from illness or disability	3 (6%)
	Caring for family	1 (2%)

### Use of Website and Exposure to Interactive Video Dramas

During the 6-month time frame, participants reported that they accessed the website at least once and on average “4-6 times”. All of the IVDs were watched by 40% of these participants; the remainder stated they had watched “some” of the scenarios.

### Responses to the Interactive Video Dramas

**Figure 1:**
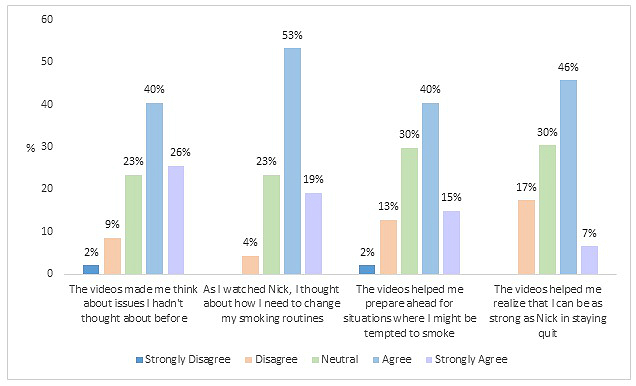
Participant responses to statements regarding the interactive video dramas.

Responses to the statement, “Nick was someone I could relate to”, indicated that the majority of participants related to the character of Nick (strongly agree=17%, agree=40%). None indicated they “strongly disagreed”, and 6 men (13%) indicated they “disagreed” that Nick was someone with whom they could relate. The remaining men (30%) rated their relatability to Nick as “neutral”. The levels of relating to Nick did not significantly differ in terms of participants’ age (*P=*.47), number of previous quit attempts in the past two years (*P=*.19), or educational background (*P=*.83). However, the participants’ level of relating to Nick was positively correlated with their overall website satisfaction (*r*_s45_=.63, *P*<.001). The majority of participants, ranging from 52-72% of the men, “agreed” or “strongly agreed” with all four statements about IVD support. Responses to each statement are shown in Figure 1.

**Figure 2:**
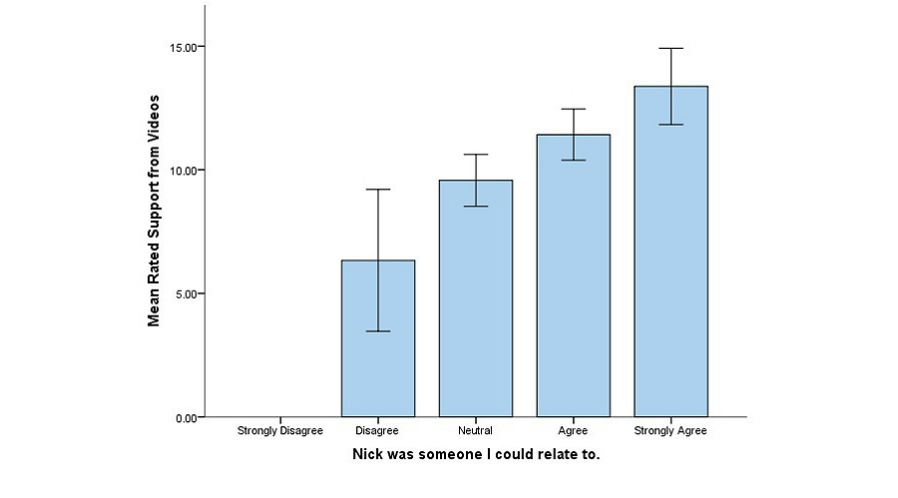
Means and standard error bars comparing relating to Nick groups on rated support from interactive video dramas.

With a possible range of 4-20, the mean IVD support score was 10.5 (SD 2.89). A statistically significant difference was found among the four levels of relating to Nick compared with perceived support from the videos (*F*_3,43_=15.181, *P*<.001). Figure 2 shows the mean IVD support score by level of relating to Nick. Post hoc Tukey HSD tests indicated that men who “strongly agreed” they could relate to Nick perceived significantly higher levels of support from the IVDs than those in the “neutral” and “disagree” groups (*P*<.001, *d*=2.0, *P*<.001, *d*=3.1) . Those in the “agree” and “neutral” groups were significantly higher on rated support from the videos than the “disagree” group (*P*<.001, *d*=2.2, *P*=.01, *d*=1.5).

## Discussion

### Principal Findings

The current study focused on examining the IVDs as one component of a multipart gender-sensitive website designed to support men’s smoking cessation. IVDs are an innovative approach in men’s health promotion research [12], and the current study findings document the first application of IVDs in smoking cessation. More specifically, it is the first to target men who smoke. Results indicate that the IVDs included in the *If I were Nick* series were perceived as supportive by many men who were interested in reducing and quitting smoking.

The finding that only 47 of the 117 potential respondents viewed at least some of the IVDs may have been influenced by the placement of the IVDs on the QuitNow Men website [13]. The IVDs could not be directly accessed from the home page (*If I were Nick* was accessible only after clicking on the tab “Tactics & Tools”) and without a visual identifier on the home page may have been missed. However, it is encouraging that almost two-thirds of the men viewed at least some of the IVDs, and among those who viewed the IVDs, 40% viewed all 7 of the scenarios, providing an indication of the acceptability of this approach to men. The potential success of IVDs, as indicated by men’s uptake of and perceived support from them, affirms the need for ongoing evaluation of the effectiveness of IVDs as they relate to smoking cessation and, more generally, men’s health promotion.

Our finding that a majority of men related to Nick suggests that it may be important to ensure that the main IVD character is someone with whom the audience can identify. It appears, though, that the character may not need to perfectly mirror the demographics of the target audience. For instance, relatability was not associated with viewers’ age or previous quit attempts. It was especially noteworthy that the character’s employment as a construction worker did not mitigate identification with the character by middle class men working in the tertiary sector. These findings may suggest that men were connecting with the struggles of Nick and his reaction to the challenges that he faced, rather than demographic identity (eg, ethnicity, class, age, marital status). The modeling and permission of “other” men is also known to deeply influence the actions and non-actions of men in relation to their health [4]. Bearing both these points in mind, the resonance of Nick with end-users may flow from *how*, as well as *what* he does—rather than *who* he is perceived to be. It is possible that Nick’s workmanlike routine and its segmented connections to smoking resonated with the participants’ desire to feel more competent and prepared to execute a quit. As such, participants could have identified not only with Nick, but with his challenges around smoking cessation—amid being prompted to choose their own strategies. So while men’s identity has often been linked to their jobs or careers, identification with Nick and the quitting challenges represented in the IVDs were less likely to depend on alignments to the end-users’ employment status or specific job.

There were some men who did not relate with Nick. This could reflect a desire to “stand out from the herd”, and dis-identify with this “ordinary” man, or perhaps claim identities that counter and contrast some aspect of Nick (masculine representation, age, class, occupation, culture). The failure to identify with Nick might also reflect participants’ uncertainty about their readiness to quit—wherein they critiqued the IVD product or its utility rather than the process of quitting depicted therein. Future qualitative research might explore the variation in the men’s identification with Nick, as well as variation in response to Nick based on age difference, given the participants in this study were on average 37 years old.

Although information on the challenges faced while quitting smoking are widely available on the Internet, the men’s responses to *If I were Nick* point to the potential merit of the IVDs in presenting this information through peer dialogue and real-life situations. The IVDs were designed to promote the internal reflection of what men may think, feel, and do, in certain situations. Men often avoid discussing emotion as it is not considered “manly” or may be misconstrued as weakness. By providing the internal dialogue of a fellow man and his struggles, the IVDs are intended to normalize feelings that often go undiscussed among the male population. These emotions are further supported through seeing the responses of other men once they have identified and locked in their responses using the reflective questions.

The IVDs were also designed to empower the audience by assisting men in visualizing and preparing for the challenges of quitting and exposing them to the requisite skills needed to sustain their quit thereby boosting self-efficacy, a critical mediating influence on smoking cessation outcomes [18,19]. The findings from the current study indicated that preparing and planning strategies, and providing choices in the context of an IVD, hold potential for enabling men to action their interest in reducing or quitting smoking. The disaggregated day’s events, so typical of many men’s lives, may have prompted participants to deconstruct specific routines and rituals common to their everyday lives while prospectively planning what they might do in similar circumstances. In an online environment, a men’s IVD smoking cessation intervention also allows for that planning and strategy work to take place in an anonymous setting, free of experts, which may be perceived by men as taking action rather than receiving help—an approach in accord with masculine values.

There is much uncertainty about best practices for IVDs in health promotion, and in the specific context of men’s smoking cessation, clearly, there is much to learn. Beyond identifying (or not) with the central character (which could be remedied through technologies enabling end-users to select an avatar or choose from a range of characters or insert their own image to a vignette), the number and duration of the IVD scenarios might influence the uptake and “completion” of the series. Herein lies the need for future large, longitudinal, comparative research to better apprehend and predict what media and mechanisms might prevail within an ever changing “virtual” forum for men’s health promotion. Future research might also measure quit rates to assess the impact of IVDs on men’s smoking cessation.

### Conclusions

IVDs are an exciting new approach to support men’s smoking cessation that provide the opportunity to combine emotional, social, cognitive, and behavioral aspects of quitting. A well-developed IVD can incorporate the principles of men’s health promotion, include multiple features that appeal to men and convey valuable smoking cessation tactics in an instructive yet empowering way. When men gain new information and believe they are skilled enough to apply it successfully, they are more likely to try and to succeed in quitting smoking. Given that the use of IVDs in health promotion is in its infancy, the positive outcomes from this study suggest the potential for IVDs and warrant moving forward in this area.

## Appendix

Multimedia Appendix 1 Meet Nick.

Multimedia Appendix 2 Morning routine.

Multimedia Appendix 3 On the road to work.

Multimedia Appendix 4 Need a break.

Multimedia Appendix 5 Out with the guys.

Multimedia Appendix 6 Stressed out.

Multimedia Appendix 7 On track.

## Abbreviations

IVD: interactive video drama
